# Sensing Performance Investigations on Two-Photon Fluorescent Probes for Detecting *β*-Amyloid in Alzheimer’s Disease

**DOI:** 10.3390/s20061760

**Published:** 2020-03-22

**Authors:** Yujin Zhang, Ni Luan, Kan Li, Jiancai Leng, Wei Hu

**Affiliations:** 1 School of Electronic and Information Engineering (Department of Physics), Network Information Center, Qilu University of Technology (Shandong Academy of Sciences), Jinan 250353, China; zhangyujin@qlu.edu.cn (Y.Z.); luanni_qlu@163.com (N.L.); likan@qlu.edu.cn (K.L.); jiancaileng@qlu.edu.cn (J.L.); 2 School of Chemistry and Pharmaceutical Engineering, Shandong Provincial Key Laboratory of Molecular Engineering, Qilu University of Technology (Shandong Academy of Sciences), Jinan 250353, China

**Keywords:** two-photon absorption, fluorescent probe, *β*-amyloid detection, Alzheimer’s disease

## Abstract

Alzheimer’s disease (AD) is one of the most common forms of senile disease. In recent years, the incidence of AD has been increasing significantly with the acceleration of the aging process of the global population. However, current clinical drugs can only alleviate the symptoms of AD patients without healing the disease fundamentally. Therefore, it is of great significance to develop an effective small molecule diagnostic reagent for the early diagnosis of AD. In this paper, we employ an integrated approach, including molecular docking simulation and quantum mechanics/molecular mechanics calculation, to investigate the sensing performance of a series of donor–acceptor structural probes for the marker protein of AD (*β*-amyloid). Results show that the probes display evident fluorescence enhancement when bound to the *β*-amyloid, suggesting the effect of the environment on the molecular properties. Especially, the two-photon absorption cross-section of the probes increase drastically in the *β*-amyloid compared to that in vacuum, which results from the larger electron delocalization and dipole moment in the fibrillary-like environment. Thus, one can propose that the studied probes are capable of application in two-photon fluorescent imaging, particularly those containing naphthalene rings as the donor or with a longer spacer group. Our calculations elucidate the experimental measurements reasonably, and further establish possible structure–property relationships that can be used to design novel biocompatible two-photon fluorescent probes for the diagnosis of Alzheimer’s.

## 1. Introduction

Alzheimer’s disease (AD) is a progressive disease, in which dementia symptoms gradually worsen over a number of years. In early stages of the disease, memory loss of the patient is mild, but at late stages, individuals will lose the ability to carry on a conversation or respond to the environment [1,2]. According to reports of the Alzheimer’s Association, AD is the sixth leading cause of death in the United States. Today, there is a worldwide effort on finding better approaches to treat the disease, delay its onset, and prevent it from developing [2]. However, it is difficult because the pathology of Alzheimer’s is not clear at present. According to the amyloid cascade hypothesis [3,4], deposits of *β*-amyloid are the hallmark of AD, which appears dozens of years before the symptoms occur [5,6]. Thus, there exists an urgent need to develop biochemical probes for the *β*-amyloid detection in order to improve the early diagnosis of Alzheimer’s [7,8].

In comparison to the probes based on radionuclide imaging techniques such as positron emission tomography (PET) and single-photon emission computed tomography (SPECT), a near-infrared fluorescent (NIRF) probe shows advantages on the safety, accuracy, sensitivity, as well as operability [9,10,11]. Nevertheless, a commonly used optical imaging approach based on one-photon excitation is often accompanied with interferences from self-absorption, auto-fluorescence, and photodamage [12,13]. In this context, the two-photon microscopy technique, which achieves increased penetration depth, small fluorescence background, and high three-dimensional resolution, has attracted increasingly more attention [14,15].

A potential route to identify efficient two-photon sensors for *β*-amyloid imaging is to explore the two-photon properties of the dyes that have already shown some success in this aspect. Up to now, plenty of fluorescent probes with multiple chemical structures have been synthesized and experimentally applied to detect the *β*-amyloid plaques in vivo, such as PAD-1, DCIP-1, and BAP-1 with donor–acceptor architecture [16,17,18], and QAD1, DADNIR-2, and CRANAD-3 with donor–acceptor–donor architecture [19,20,21]. Due to some correlations between the donor–π–acceptor structural compounds and their optical properties that have already been established [22,23], we choose molecules with the *N,N*-dimethylamino group as the donor and dicyano group as the acceptor to study their possibility to be two-photon fluorescent probes for detecting *β*-amyloid.

In the past decades, several dicyano-containing dyes have been designed as NIRF probes for *β*-amyloid imaging. Ono et al. selected benzothiazole as the highly polarized bridge and connected *N,N*-dimethylamino and dicyanomethylene to synthesize a series of push–pull dyes [24]. In binding experiments in vitro, the probe PP-BAT-1 showed a high affinity for *β*-amyloid aggregates. Before long, they evaluated the feasibility of a series of smart NIRF probes with donor–acceptor structure bridged by a conjugated π-electron chain for detecting *β*-amyloid [25]. The probe PP-BAT-3 with high affinity was selected for in vivo imaging and showed excellent application prospects. In addition, Fu et al. reported a series of novel probes that displayed a high sensitivity and affinities to *β*-amyloid plaques by replacing the benzene ring with a naphthalene ring, which could readily penetrate the blood–brain barrier with a large initial brain uptake [26].

**Figure 1:**
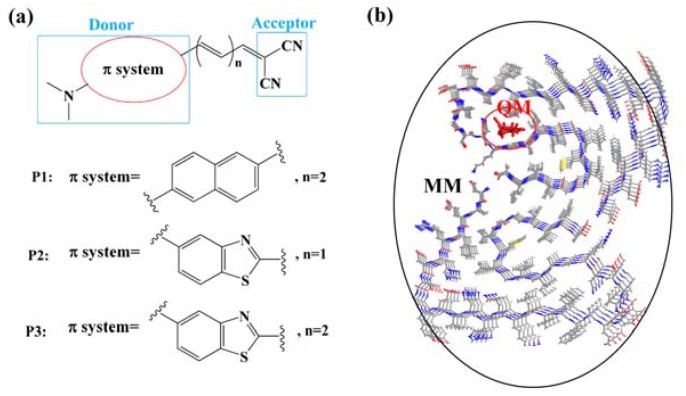
(**a**) Molecular structures of P1, P2, P3, and (**b**) the division of quantum mechanics (QM) and molecular mechanics (MM) regions in amyloid-ligand configuration.

Molecular structures of the probes studied in this paper (hereafter referred to as P1, P2, and P3) are shown in Figure 1a. Even though the probes investigated herein have been reported as successful diagnostic agents for fluorescence imaging toward *β*-amyloid [26,27], our motivation lies in characterizing the potential of these molecules to be two-photon probes for *β*-amyloid in Alzheimer’s disease. Additionally, we aim to set out possible structure–property relationships for the future design of improved diagnostic probes by analyzing the effect of donor moieties and the length of spacers on the sensing performance of the probes. To this end, another three derivatives of the same series named P4, P5, and P6 (see Figure S1) are investigated, and only the first three with a higher affinity are reported in this text. The binding profile, as well as the *β*-amyloid-specific optical properties, will be discussed in detail by employing the combined molecular docking simulation and quantum mechanics/molecular mechanics (QM/MM) calculations.

## 2. Computational Details

### 2.1. Molecular Docking

Molecular docking was carried out to identify the potential binding sites of P1, P2, and P3 with *β*-amyloid. The geometry for the probes was optimized by the density functional theory (DFT) method at the B3LYP/6-31+G(d,p) level using Gaussian 16 [28]. A full-length amyloid beta structure with a high resolution of 4 Å (PDB code 5OQV) was obtained by cryo-electron microscopy experiments [29]. As there are usually multiple binding sites for a probe in *β*-amyloid and no well-defined binding site reported previously, we employ blind docking studies with a grid box of 126 × 126 × 76 points and a spacing of 0.464 Å in AUTODOCK 4.2 software [30]. The semi-flexible approach was chosen, in which the protein was fixed and the ligands were free to move during the docking simulation. The Lamarckian genetic algorithm was chosen for ligand conformation search [31] and the other parameters kept the default settings. The simulation generated 500 docking modes with high binding affinity. Then, clustering of the docking solutions was carried out and the amyloid-ligand complexes with the lowest binding free energy for each binding mode were picked up for further analysis.

### 2.2. Property Calculations

Amyloid-ligand configurations were optimized using the QM/MM method with the two-layer ONIOM approach [32]. The ligand was regarded as the high layer and handled by the QM method, while the *β*-amyloid was treated as the MM part and defined to be the low layer (see Figure 1b). When performing ONIOM calculations, the universal force field (UFF) was adopted for the MM part and the B3LYP functional with the 6-31+G(d,p) basis set was chosen for the QM part. Besides, the electronic embedding scheme was used in the QM/MM calculations, where the wave function of the QM part was polarized by the MM point charges [33]. The MM part was frozen during the geometry optimizations, and frequency calculations were then performed to verify the stability of the optimized structures.

The amyloid-specific one-photon absorption (OPA) and emission (OPE) spectra of the probes in different binding sites of *β*-amyloid were acquired using the same QM/MM approach, as mentioned above. All these calculations were performed in the Gaussian16 package.

Last but not least, two-photon absorption (TPA) properties for the probes were calculated by employing the response theory method with the DFT/B3LYP functional and 6-31G+(d,p) basis set using Dalton2013 software [34]. In order to simulate the fibrillar environment, the polarizable continuum model was used to model the solvent effect of water [35].

## 3. Results and Discussion

### 3.1. Binding Sites for the Probes with β-Amyloid

**Figure 2:**
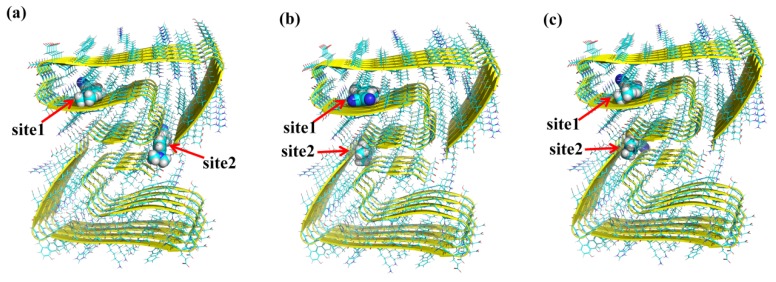
Binding sites of (**a**) P1, (**b**) P2, and (**c**) P3 in *β*-amyloid.

**Figure 3:**
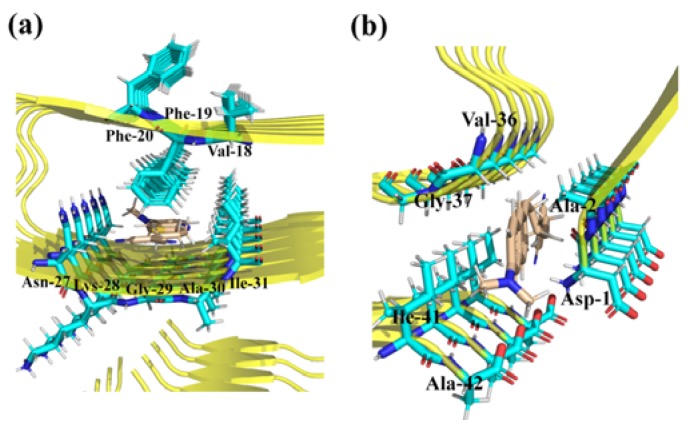
Binding details of P1 in (**a**) site1 and (**b**) site2 in *β*-amyloid.

Molecular docking simulations on the probes show that there are two most likely binding sites for each probe toward *β*-amyloid, as demonstrated in Figure 2 and Figure S2. Both binding sites for P1, P2, and P3 are inside the fibril, which might be dominated by the volume of the locus [36]. In order to obtain deeper insights into the amyloid-ligand binding profile, different binding modes of P1 in *β*-amyloid are shown in Figure 3 as an example. The probe inserts into binding site1 formed by Val-18, Phe-19, Phe-20, Asn-27, Lys-28, Gly-29, Ala-30, and Ile-31, while in binding site2, the probe is surrounded by Asp-1, Ala-2, Gly-37, Ile-41, and Ala-42. Careful inspection of the residues shows that only Phe-19 in site1 is aromatic ring-contained, which is favorable to have π–π interactions with the ligand. As a result, the binding energy of site1 is much lower than that of site2 for all probes (see Table 1). Compared to the binding free energies for P1 in site1 (−12.54 kcal/mol) and site2 (−8.09 kcal/mol), the values for P2 increases to −10.54 and −6.98 kcal/mol, indicating that the substitution from benzothiazole to naphthalene can effectively enhance the affinity of the probe to the *β*-amyloid. Furthermore, in comparison to P2, probe P3 with a longer polyenic chain possesses a lower binding free energy. Thus, the length of the π-conjugated bridge between the donor and acceptor units also contributes to molecular binding affinity.

Apart from the binding energy, the inhibition constant is another important parameter to evaluate the bioactivity of an agent. The calculated data from molecular docking are listed in Table 1 and Table S1. It can be seen that the inhibition constants of P1 in site1 and site2 are 0.645 and 118 nM, respectively, while those for P2 and P3 are 18.86 and 767 nM, and 2.86 and 166 nM, respectively. This verifies that site1 is the one associate with a stronger binding affinity. The free energy of binding for different probes follows the sequence P1 > P3 > P2. Note that the experimental binding constants for P1, P2, and P3 are reported to be respectively 96.7, 148, and 40.1 nM [26,27], suggesting it may be an average over various binding sites.

### 3.2. β-Amyloid-Specific OPA Properties

**Figure 4:**
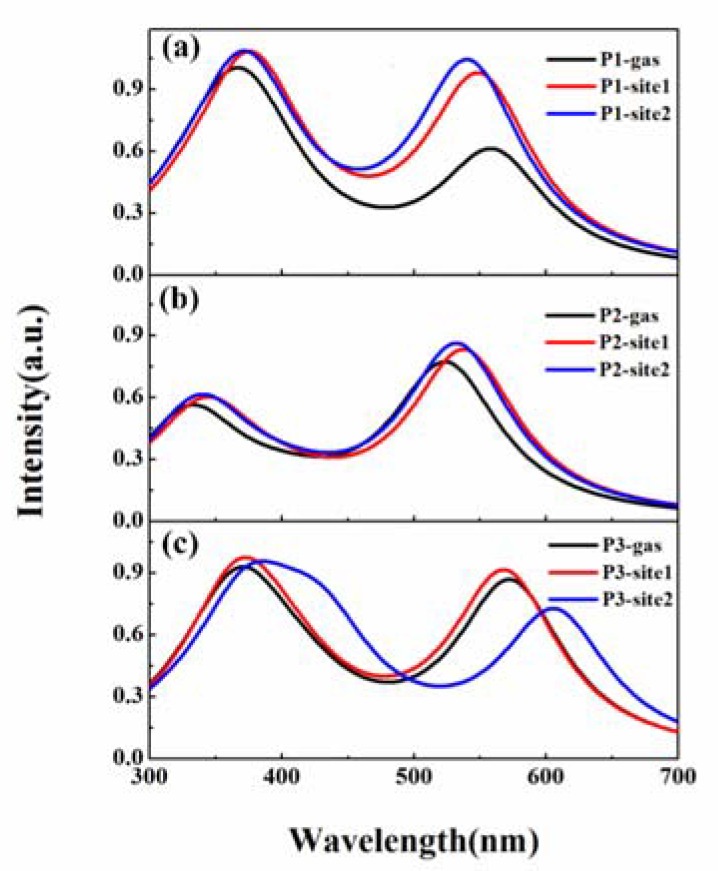
One-photon absorption (OPA) spectra for (**a**) P1, (**b**) P2, and (**c**) P3 in different microenvironments.

In view of multiple binding modes found for the probes toward *β*-amyloid, one can expect that molecular photophysical properties may feature a combined effect over contributions from different sites. We computed OPA spectra for the probes in different binding sites, and the spectrum in vacuum is also shown as a reference in Figure 4. Obviously, there are two absorption bands with high intensity for P1, P2, and P3 both in the gas phase and in *β*-amyloid, which are located at wavelengths around 350 and 550 nm, respectively. 

**Figure 5:**
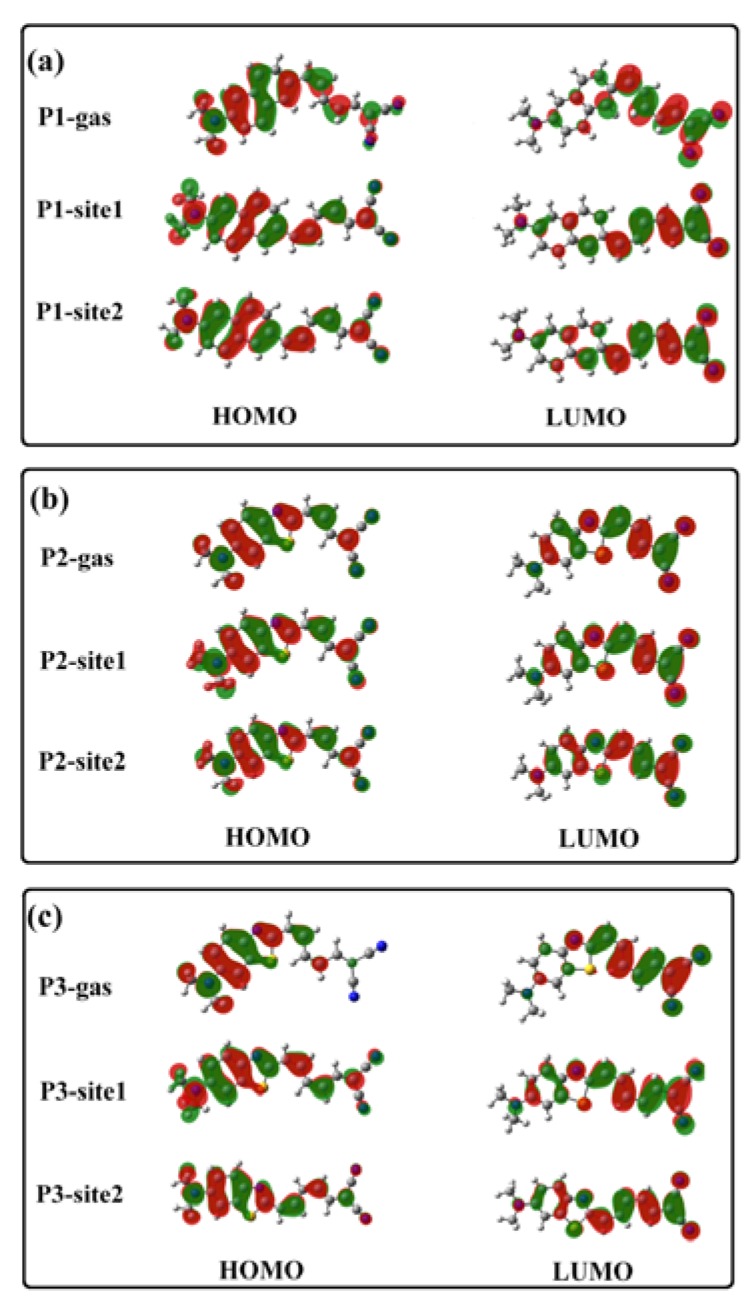
HOMO and LUMO for (**a**) P1, (**b**) P2, and (**c**) P3 in the ground state in different microenvironments.

An analysis of molecular orbitals involved in the OPA process shows that the absorption band in the long wavelength range is associated with the transition from the highest occupied molecular orbital (HOMO) to the lowest unoccupied molecular orbital (LUMO) of a probe. Frontier molecular orbitals for the probes in different microenvironments are shown in Figure 5. Evidently, the HOMO to LUMO transition shows a charge transfer characteristic from the donor moiety to the acceptor part.

Details of the OPA peak with a wavelength larger than 500 nm for the probes, including absorption wavelength, the corresponding oscillator strength, and transition nature, are listed in Table 2 and Table S2. In vacuum, the OPA maxima are 560 and 524 nm for P1 and P2, respectively, showing a blueshift when replacing the naphthalene ring in P1 with the benzothiazole moiety. However, the maximum absorption peak for P3 is redshifted to 573 nm in comparison to the spectra of P1 and P2. In *β*-amyloid, the absorption wavelength of P1 is blueshifted by 11 and 19 nm in binding site1 and site2, respectively, compared to the case in gas, indicating the microenvironment effect on the molecular optical properties. On the contrary, the maximum OPA peaks of P2 and P3 are redshifted in *β*-amyloid, especially for P3 in site2, and the shift is more than 30 nm. Thus, adding a double bond to the dye skeleton may result in a redshift of the maximal OPA wavelength of these probes. As it has been reported, the experimental absorption band maxima are 557 nm for P2 and 559 nm for P3 [27]. Even though there is a quantitative difference between the experimental measurement and the computed values, the decrease trend in excitation energy with a larger spacer length is reproduced satisfactorily.

In addition to the shifts in OPA spectra, the absorption intensity for the probes is increased to different degrees when bound to *β*-amyloid, except for the case of P3 binding in site2. According to the equation $\delta_{OPA}=(2\omega_{f}{\sum_{\alpha }\left|\langle i|\mu_{\alpha }|f\rangle \right|}^{2})/3$ [37], where *ω_f_* and *μ_α_* denote the excited energy of the state |*f* > and electric dipole moment operator in the direction *α*, respectively, OPA strength is dominated by the transition dipole moment between the ground state and the excited state. Calculated transition dipole moments for P1, P2, and P3 are 3.17, 3.56, and 3.91 Debye in vacuum, respectively. Within *β*-amyloid, molecular transition dipole moments are increased for P1 and P2 both in site1 and in site2. However, for P3 in site2, the value is reduced to 3.63 Debye compared to that in gas, resulting in a smaller oscillator strength.

### 3.3. β-Amyloid-Specific OPE Properties

**Figure 6:**
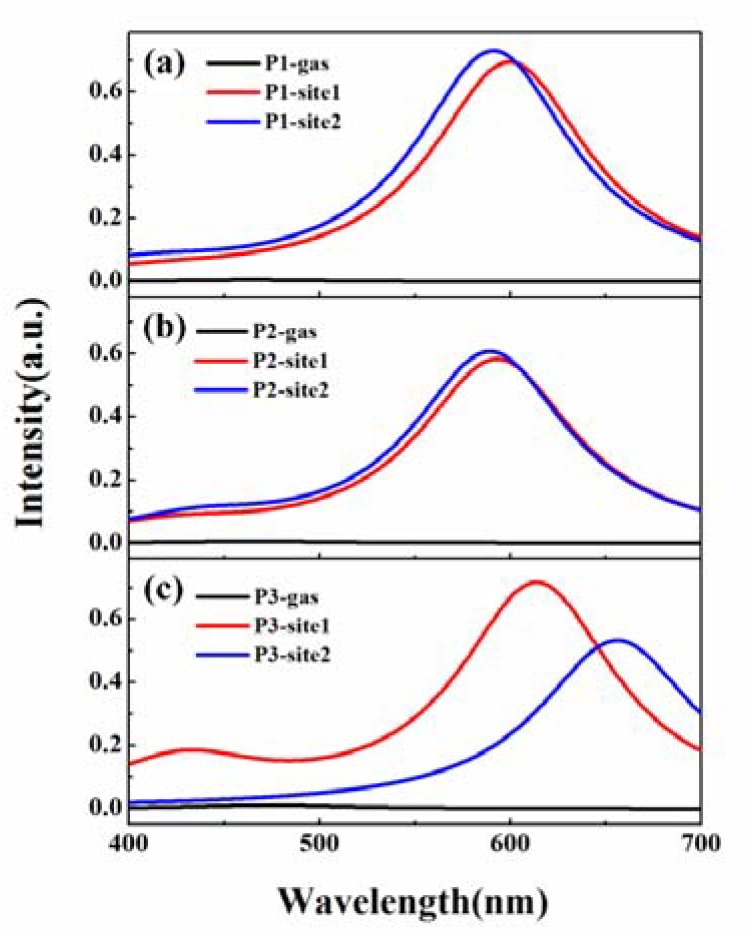
One-photon emission (OPE) spectra for (**a**) P1, (**b**) P2, and (**c**) P3 in different microenvironments.

To investigate the feasibility for the dyes to detect *β*-amyloid as a fluorescent sensor, OPE properties of the probes in different microenvironments are investigated by optimizing the molecular geometry in the first excited state using the QM/MM method. One can see from the emission spectra in Figure 6 and Table S3 that the fluorescence of the probes is quenched in the gas phase, while it is increased significantly in *β*-amyloid. This off–on transformation on the fluorescent signal of the probes appears to be in good agreement with the experimental observations. Within *β*-amyloid, the maximum emission wavelengths for P1 in site1 and site2 are respectively 600 and 591 nm. By replacing the donor moiety with benzothiazole in P1, the emission maxima are shifted to 593 nm (589 nm) for P2 and 614 nm (656 nm) for P3 in site1 (site2). Although these values are smaller than the experimental results of 660 nm for P2 and 710 nm for P3 [27], the lengthened trend of emission wavelength for the push–pull-type dyes by increasing the number of conjugated double bonds is reproduced well (see Table S3) [38]. In general, the numerical discrepancy can be attributed to the flexibility of the *β*-amyloid and nuclear motion of the probe that are not included in our treatment.

**Figure 7:**
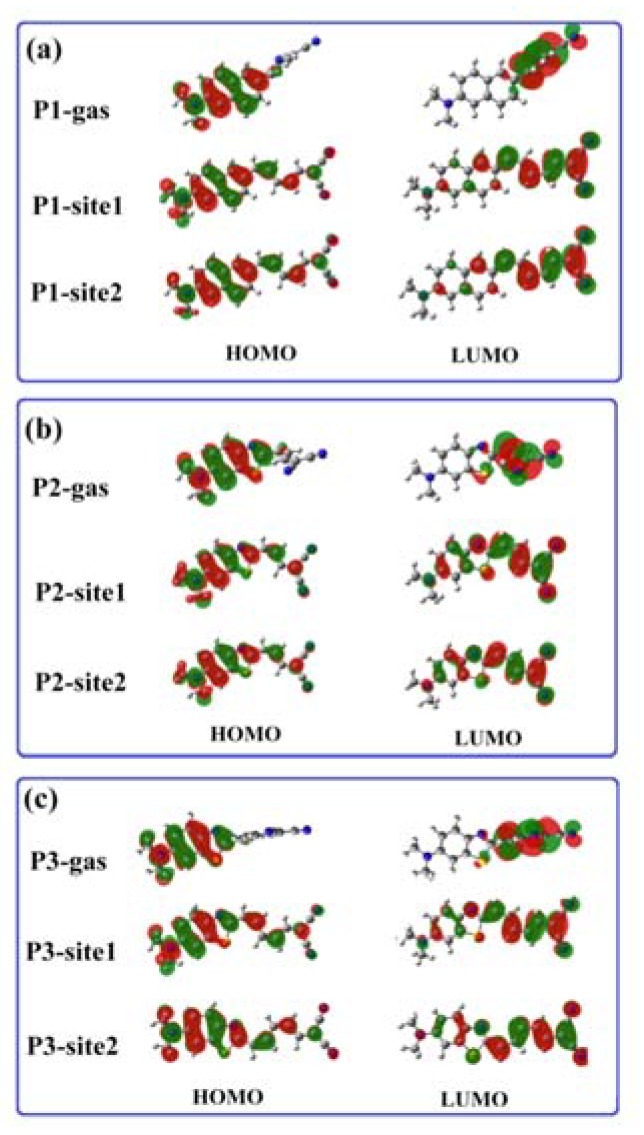
HOMO and LUMO for (**a**) P1, (**b**) P2, and (**c**) P3 in the first excited state in different microenvironments.

Transition properties of the first excited state play an important role in determining the fluorescent characteristics. Thus, analysis of the nature of excitation corresponding to the emission peak is performed. It shows that the fluorescence of the probes in amyloid comes from the molecular first excited state radiation, which is predominated by the LUMO-to-HOMO transition. As shown in Figure 7, HOMOs and LUMOs of P1, P2, and P3 in the gas phase are mainly localized in the donor and acceptor moiety, respectively. This separate distribution generates a small overlap on the orbitals, which results in the non-fluorescent feature of the probes. Unlike the case in vacuum, frontier molecular orbitals for the probes in amyloid are extended on the whole molecule. Thus, the oscillator strength of the molecular first excited states is enhanced (see Table S3) and fluorescence is recovered.

**Figure 8:**
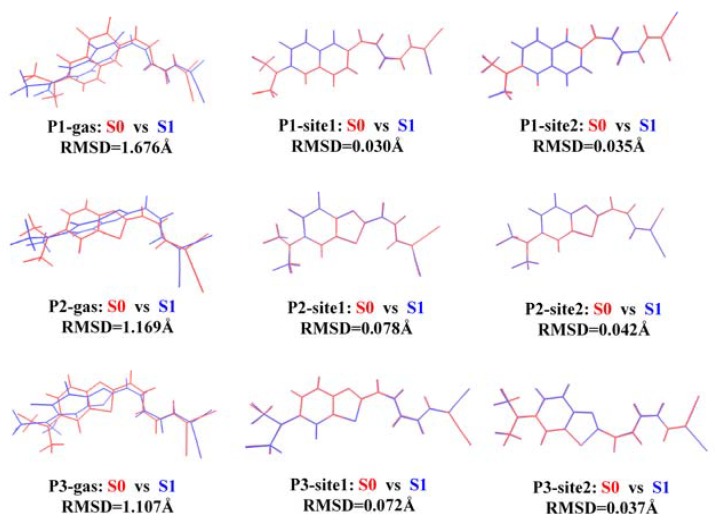
Geometry changes between the ground state and the first excited state for P1, P2, and P3 in different microenvironments.

In order to rationalize the changes in molecular orbitals’ distribution, geometries of the probes both in gas and in *β*-amyloid are displayed in Figure 8. It is apparent that the dihedral angle between the donor and acceptor of P1, P2, and P3 is largely increased upon excitation. Consequently, the donor and acceptor units are almost perpendicular to each other, leading to weak charge transfer between them and small emission intensity. In *β*-amyloid, changes in the geometries between the ground state and the first excited state of the probes are not obvious, because the rotation of the groups is hindered by the amyloid. To quantitatively characterize the geometric variations between the ground state and the first excited state of the probes, the root-mean-square displacement (RMSD) for the two states in different microenvironments is calculated using Multiwfn [39]. In the gas phase, the RMSD for P1, P2, and P3 are 1.676, 1.169 and 1.107 Å, respectively. When bound to *β*-amyloid, the values are smaller than 0.1 Å, showing a slight change in the molecular configuration in different states. In conclusion, the probes show off–on fluorescence when they are bound to the amyloid, indicating the availability of P1, P2, and P3 as fluorescent probes for detecting *β*-amyloid in Alzheimer’s disease.

### 3.4. β-Amyloid-Specific TPA Properties

Motivated by the results above, we next investigate the probability of the probes as a two-photon fluorescent sensor for *β*-amyloid imaging. The macroscopic TPA cross-section (*σ_TPA_*) that can be directly compared to the experiment is defined as [40]

(1) $$\sigma_{TPA}=\frac{4\pi^{2}a_{0}^{5}\alpha }{15c}\frac{\omega^{2}g(\omega )}{\Gamma_{f}}\delta_{TPA}$$

Here, *a*_0_ is the Bohr radius, *c* is the speed of light, *α* is the fine structure constant, *ω* is the frequency of the incident light, and *g*(*ω*) denotes the spectral line profile, which is usually assumed to be a delta function. The level broadening Γ*_f_* of the final state is commonly assumed to be 0.1 eV, corresponding to a lifetime of about 6 fs. *δ_TPA_* in the above equation denotes the microscopic TPA cross-section. When excited by linearly polarized light, the expression of *δ_TPA_* can be written as [41]

(2) $$\delta_{TPA}=\sum_{\alpha \beta }\left[2\times S_{\alpha \alpha }\times S_{\beta \beta }^{*}+2\times S_{\alpha \beta }\times S_{\alpha \beta }^{*}+2\times S_{\alpha \beta }\times S_{\beta \alpha }^{*}\right]$$

where the two-photon transition matrix element in the case of resonant degenerate TPA can be expressed by [42]

(3) $$S_{\alpha \beta }=\sum_{i}\left[\frac{\langle 0|\mu_{\alpha }|i\rangle \langle i|\mu_{\beta }|f\rangle }{\omega_{i}-\omega_{f}/2}+\frac{\langle 0|\mu_{\beta }|i\rangle \langle i|\mu_{\alpha }|f\rangle }{\omega_{i}-\omega_{f}/2}\right]$$

Here, the summation goes over all intermediate states |*i*> including the ground state |0> and the final state |*f*>, *ω_ι_* is the corresponding intermediate state energy, and *μ_α(β)_* is the dipole moment operator in the direction *α,β* ∈ (*x, y, z*).

Calculated TPA cross-sections for the probes both in the gas phase and in different binding sites within *β*-amyloid are presented in Table 3 and Table S4. It is worth highlighting that the TPA cross-section for the probes in amyloid is more than two times larger than that in the gas phase, which reveals a substantial environmental effect on the molecular nonlinear optical properties. This also suggests that P1, P2, and P3 can be used as promising two-photon probes in Alzheimer’s early diagnosis. The TPA cross-section maxima decrease from about 1000 to about 350 GM in *β*-amyloid (411 to 166 GM in the gas phase) going from P1 to P2, showing the influence of donor moieties on the two-photon response of the probes. However, the value of the maximum TPA cross-section possesses a nearly threefold enhancement in P3 compared to P2, corresponding to an increase in the TPA performance upon spacer elongation. Particularly, the maximal TPA wavelength for the probes is located in the near-infrared region, which indicates the possibility of probing a deeper region in practice. Moreover, the TPA wavelength maximum of the probes is comparable to the value of 1000 nm for an experimentally reported two-photon *β*-amyloid sensor [43], which confirms the reasonability of our calculations.

As is known, the TPA cross-section is dominated by the two-photon transition matrix element. Here, we set the long axis of a molecule as the *x* direction and short axis of the molecule as the *y* direction. As can be seen in Table 3, the maximum TPA cross-section of the probes is mainly contributed by the two-photon transition matrix element *S_xx_* in different microenvironments, which corresponds to the transition along the molecular macroaxis. When the probes are bound to *β*-amyloid, the value of *S_xx_* increases sharply, indicating an enhancement in transition dipole moment between the ground state and the TPA state of the probe according to Equation (3). Moreover, the magnitude of *S_xx_* for different probes follows the order P1 > P3 > P2 both in vacuum and in *β*-amyloid, showing the same trend as the maximum TPA cross-section. One can thus achieve the conclusion that the substitution of the benzothiazole moiety or lengthening of the π bridge in P2 would effectively improve the electron delocalization and transition dipole moment of the molecule, further enhancing the TPA transition probability. The above theoretical investigations reveal the inner perspectives for the usage assumption on the probes to be a practicable two-photon imaging chemosensor for detecting *β*-amyloid plaques in Alzheimer’s disease.

## 4. Conclusions

Encouraged by the good qualities of two-photon excited fluorescent imaging, we have studied a series of donor–acceptor dyes for their usage in Alzheimer’s early diagnosis as a two-photon fluorescent probe. The binding profiles and photophysical properties of the probes in *β*-amyloid are analyzed by using an integrated approach including molecular docking and QM/MM calculations. Results demonstrate that these probes show evident fluorescence enhancement when bound to the amyloid, indicating the key role of the environmental effect on molecular properties. Furthermore, an increased molecular TPA cross-section is found in the *β*-amyloid compared to that in the gas phase, which results from the large electron delocalization and dipole moment. We arrive at the conclusion that the studied probes can be applied for the two-photon imaging technique, particularly the ones containing a naphthalene ring as the donor and with a longer spacer group. Our calculations give a reasonable explanation for the previous experimental observations, and provide information on the molecular structure–property relationship, which is beneficial for developing new efficient fluorescent probes toward *β*-amyloid.

## Figures and Tables

**Table 1:** Energy parameters (in kcal/mol) and inhibition constant (in nM) for P1, P2, and P3 in various binding sites in *β*-amyloid.

Site	P1		P2		P3	
	Site1	Site2	Site1	Site2	Site1	Site2
Binding energy	−12.54	−8.09	−10.54	−6.98	−11.66	−7.89
Inhibition constant	0.645	118	18.86	767	2.86	166
Intermolecular Energy	−13.73	−9.28	−11.43	−7.87	−12.85	−9.08
Internal Energy	−0.46	−0.43	−0.44	−0.44	−0.56	−0.56
Torsional Energy	1.19	1.19	0.89	0.89	1.19	1.19
Unbound Energy	−0.46	−0.43	−0.44	−0.44	−0.56	−0.56

**Table 2:** OPA wavelength *λ_OPA_*(nm), oscillator strength *δ_OPA_*(a.u.), transition nature, and the dipole moment *μ*(Debye) for P1, P2, and P3 in different microenvironments in long wavelength region. H(L) denotes HOMO(LUMO).

Site	P1			P2			P3		
	Gas	Site1	Site2	Gas	Site1	Site2	Gas	Site1	Site2
*λ_OPA_*	560	549	541	524	538	533	573	568	606
*δ_OPA_*	0.55	0.90	0.96	0.73	0.79	0.82	0.81	0.85	0.66
Transition Nature	H-L 98%	H-L 98%	H-L 98%	H-L 98%	H-L 98%	H-L 98%	H-L 98%	H-L 98%	H-L 98%
***μ***	3.17	4.03	4.14	3.56	3.75	3.80	3.91	3.99	3.63

**Table 3:** The maximum two-photon absorption (TPA) wavelength *λ_TPA_*(nm), TPA cross-section *σ_TPA_* (GM, 1 GM = 10^−50^ cm^4^ · s/photon), and the corresponding two-photon transition matrix element *S_αβ_* for P1, P2, and P3 in different microenvironments.

Site	*λ_TPA_*	*σ_TPA_*	*S_xx_*	*S_yy_*	*S_zz_*	*S_xy_*	*S_xz_*	*S_yz_*
P1-gas	1119	411	757.9	2.8	0.8	15.8	10.6	2.3
P1-site1	1206	1099	1335.2	1.1	1.1	43.6	7.5	0.2
P1-site2	1183	1088	1303.1	0.3	1.1	33.9	15.0	0.6
P2-gas	1048	166	449.1	3.9	1.1	30.0	0.0	0.0
P2-site1	1129	338	690.9	5.6	1.6	40.4	1.5	0.8
P2-site2	1140	366	726.0	4.0	1.4	36.0	4.2	0.3
P3-gas	1080	344	664.4	10.2	0.9	34.7	0.0	0.0
P3-site1	1224	894	1222.0	3.3	1.5	23.4	9.6	0.6
P3-site2	1281	934	1308.8	2.8	1.4	33.6	0.2	0.1

## Supplementary Materials

The following are available online at https://www.mdpi.com/1424-8220/20/6/1760/s1, Figure S1: Molecular structures of P4, P5 and P6, Figure S2: Binding sites of (a) P4, (b) P5, (c) P6 in *β*-amyloid, Table S1: Energy parameters (in kcal/mol) and inhibition constant (in nM) for P4, P5 and P6 in various binding sites in *β*-amyloid, Table S2: OPA wavelength λ_OPA_(nm), oscillator strength δ_OPA_(a.u.) and the corresponding transition nature for P1, P2, P3, P4, P5 and P6 in different microenvironments at long wavelength region. H(L) donates HOMO(LUMO), Table S3: OPE wavelength λ_OPE_(nm), oscillator strength δ_OPE_(a.u.) and the corresponding transition nature for P1, P2, P3, P4, P5 and P6 in different microenvironments at long wavelength region. H(L) donates HOMO(LUMO), Table S4: The maximum TPA wavelength *λ_TPA_* (nm), TPA cross section *σ_TPA_* (GM, 1 GM = 10^−50^ cm^4^ · s/photon), and the corresponding two-photon transition matrix element *S_αβ_* for P1, P2, P3, P4, P5 and P6 in different microenvironments.
