# Supplementary material for: Sensing Performance Investigations on Two-Photon Fluorescent Probes for Detecting β-Amyloid in Alzheimer’s Disease
Source: Sensors (Basel). 2020 Mar 22;20(6):1760. doi: 10.3390/s20061760 (PMC7146205; doi:10.3390/s20061760)
Supplement: Supplementary file 1 [file sensors-20-01760-s001.pdf]

Article

Sensing Performance Investigations on Two-Photon Fluorescent Probes for Detecting *β*-Amyloid in Alzheimer's Disease

Yujin Zhang [1], Ni Luan [1], Kan Li [1], Jiancai Leng [1] and Wei Hu [2,*]

[1] School of Electronic and Information Engineering (Department of Physics), Network Information Center, Qilu University of Technology (Shandong Academy of Sciences), Jinan 250353, China; zhangyujin@qlu.edu.cn(Y.Z.), luanni_qlu@163.com(N.L.), likan@qlu.edu.cn(K.L.), jiancaileng@qlu.edu.cn(J.L.)

[2] School of Chemistry and Pharmaceutical Engineering, Shandong Provincial Key Laboratory of Molecular Engineering, Qilu University of Technology (Shandong Academy of Sciences), Jinan 250353, China

* Correspondence: weihu@qlu.edu.cn

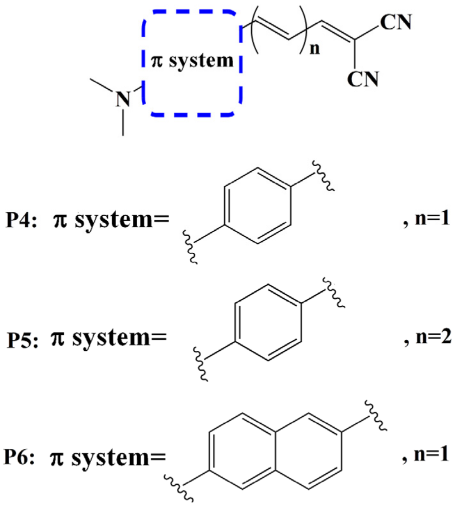

Figure 1. Molecular structures of P4, P5 and P6.

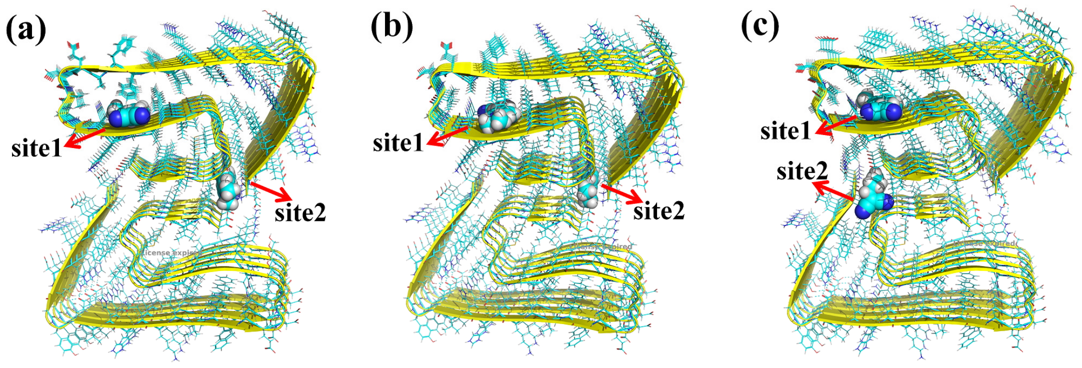

Figure 2. Binding sites of (a) P4, (b) P5, (c) P6 in *β*-amyloid.

Table S1. Energy parameters (in kcal/mol) and inhibition constant (in nM) for P4, P5 and P6 in various binding sites in β-amyloid.

Site	P4		P5		P6	
	site1	site2	site1	site2	site1	site2
Binding energy	-9.27	-6.44	-10.07	-6.77	-11.1	-8.12
Inhibition constant	160.77	18930	41.45	10890	7.33	1120
Intermolecular Energy	-10.16	-7.34	-11.26	-7.96	-11.99	-9.01
Internal Energy	-0.4	-0.41	-0.49	-0.43	-0.32	-0.59
Torsional Energy	0.89	0.89	1.19	1.19	0.89	0.89
Unbound Energy	-0.4	-0.41	-0.49	-0.43	-0.32	-0.59

Table S2. OPA wavelength λ_{OPA}(nm), oscillator strength δ_{OPA}(a.u.) and the corresponding transition nature for P1, P2, P3, P4, P5 and P6 in different microenvironments at long wavelength region. H(L) donates HOMO(LUMO).

Site	P1			P2(Exp. 557)			P3(Exp. 559)		
	gas	site1	site2	gas	site1	site2	gas	site1	site2
λ_{OPA}	560	549	541	524	538	533	573	568	606
δ_{OPA}	0.55	0.90	0.96	0.73	0.79	0.82	0.81	0.85	0.66
Transition Nature	H-L 98%	H-L 98%	H-L 98%	H-L 98%	H-L 98%	H-L 98%	H-L 98%	H-L 98%	H-L 98%
	**P4(Exp. 489)**			**P5(Exp. 519)**			**P6**		
Site	**gas**	**site1**	**site2**	**gas**	**site1**	**site2**	**gas**	**site1**	**site2**
λ_{OPA}	441	455	439	498	498	509	508	503	501
δ_{OPA}	0.71	0.89	1.05	0.81	0.90	1.06	0.48	0.97	0.80
Transition Nature	H-L 98%	H-L 98%	H-L 98%	H-L 98%	H-L 98%	H-L 98%	H-L 98%	H-L 98%	H-L 98%

Table S3. OPE wavelength λ_{OPE}(nm), oscillator strength δ_{OPE}(a.u.) and the corresponding transition nature for P1, P2, P3, P4, P5 and P6 in different microenvironments at long wavelength region. H(L) donates HOMO(LUMO).

Site	P1(Exp. 678)			P2(Exp. 660)			P3(Exp. 710)		
	gas	site1	site2	gas	site1	site2	gas	site1	site2
λ_{OPA}	859	600	591	892	593	589	924	614	656
δ_{OPA}	0	0.69	0.73	0	0.58	0.60	0	0.71	0.53
Transition Nature	L-H 98%	L-H 98%	L-H 98%	L-H 98%	L-H 98%	L-H 98%	L-H 98%	L-H 98%	L-H 98%
	**P4(Exp. 577)**			**P5(Exp. 665)**			**P6(Exp. 615)**		
Site	**gas**	**site1**	**site2**	**gas**	**site1**	**site2**	**gas**	**site1**	**site2**
λ_{OPA}	725	493	471	769	574	552	801	598	589
δ_{OPA}	0	0.67	0.83	0	0.54	0.77	0	0.41	0.39
Transition Nature	L-H 98%	L-H 98%	L-H 98%	L-H 98%	L-H 98%	L-H 98%	L-H 98%	L-H 98%	L-H 98%

Table S4. The maximum TPA wavelength λ_{TPA}(nm), TPA cross section σ_{TPA}(GM, 1GM=10^{-50} cm^4×s/photon), and the corresponding two-photon transition matrix element $S_{\alpha\beta}$ for P1, P2, P3, P4, P5 and P6 in different microenvironments.

Site	λ_{TPA}	σ_{TPA}	S_{xx}	S_{yy}	S_{zz}	S_{xy}	S_{xz}	S_{yz}
P1-gas	1119	411	757.9	2.8	0.8	15.8	10.6	2.3
P1-site1	1206	1099	1335.2	1.1	1.1	43.6	7.5	0.2
P1-site2	1183	1088	1303.1	0.3	1.1	33.9	15.0	0.6
P2-gas	1048	166	449.1	3.9	1.1	30.0	0.0	0.0
P2-site1	1129	338	690.9	5.6	1.6	40.4	1.5	0.8
P2-site2	1140	366	726.0	4.0	1.4	36.0	4.2	0.3
P3-gas	1080	344	664.4	10.2	0.9	34.7	0.0	0.0
P3-site1	1224	894	1222.0	3.3	1.5	23.4	9.6	0.6
P3-site2	1281	934	1308.8	2.8	1.4	33.6	0.2	0.1
**Site**	λ_{TPA}	σ_{TPA}	S_{xx}	S_{yy}	S_{zz}	S_{xy}	S_{xz}	S_{yz}
P4-gas	1039	238	2.2	8.6	533.2	4.1	12.3	20.3
P4-site1	962	136	1.7	4.4	372.5	0.8	5.1	15.0
P4-site2	933	155	1.9	1.7	387.6	0.9	0.5	7.2
P5-gas	1200	668	1.7	7.4	1035.2	2.7	17.9	13.6
P5-site1	1048	441	2.3	2.7	735.1	0.5	4.3	3.3
P5-site2	1085	356	2.0	2.7	684.1	0.5	2.1	0.4
P6-gas	1195	647	1.9	1.6	1015.9	7.7	10.7	3.6
P6-site1	1052	538	1.2	0.2	813.9	0.9	3.6	37.2
P6-site2	1061	409	0.8	4.8	715.3	2.8	17.1	45.9
